# Supplementary material for: Lymphatic embolization for early post-operative lymphatic leakage after radical cystectomy for bladder cancer
Source: PLoS One. 2024 Sep 24;19(9):e0305240. doi: 10.1371/journal.pone.0305240 (PMC11421775; doi:10.1371/journal.pone.0305240)
Supplement: Attachment — Submitted filename: Response to Reviewers.docx [file pone.0305240.s001.docx]

Revision Letter

Dear Editor,

We are grateful to have the opportunity to revise our manuscript titled “Lymphatic embolization for early post-operative lymphatic leakage after radical cystectomy for bladder cancer”.

We have revised the manuscript in accordance to the reviewers’ comments. We detailed the revisions that we have made below, addressing each comment. We sincerely hope that we have adequately addressed both the reviewers and your concerns and comments in our revised manuscript.

Thank you for your kind attention.

Best regards,

Ji Eun Heo, M.D.

**Reviewer #1:**

We would like to thank you for your thorough evaluation of our paper. We revised our paper after reviewing your recommendations for revision:

1. Introduction: When discussing LND extent in RCx, it would be beneficial to mention the results of the SWOG 1011 trial.

We added the SWOG 1011 trial information in our introduction, line 81-84.

2. Introduction: I recommend reporting further details on the management of lymphatic leakage in “bladder cancer” patients instead of presenting general data.

We definitely agree with your comment. Despite lymphatic complications frequently occurring up to ~10% in RC patients, not much research has been conducted on the disease course of lymphatic leakages specific to RC. We therefore believe that our study is one of the first to identify lymphatic leaks specifically related to bladder cancer. We elaborated the management of leakage specific to bladder cancer in line 84-87.

3. Methods: Were all cystectomies performed with curative intent, or were there any palliative cases?

Yes, all radical cystectomies were performed with curative intent without any palliative cases. We have specified it in line 101-102.

4. Methods: Please add details about LND. What methods were used for ligating the lymphatics (metal clips, bipolar, etc.)? How many drains in which locations were placed after the surgery?

Thank you for the suggestion. We included the information regarding the methods used to seal the lymphatics in line 128-130.

5. Methods: Daily drainage < 300 mL/day has been the indication for drain removal. Please comment on this, as it is on the high side.

As mentioned in line 168-171, the timing of catheter removal was dependent on the surgeon in charge of the patient. 300mL/day was the common indication for drainage catheter removal in our hospital. We have rephrased the paragraph for clarity.

6. Methods: The primary and secondary outcomes should be clearly defined.

We clarified our primary and secondary outcomes in 108-110. Thank you for the suggestion.

7. Results: Given the number of cases, the continuous variables should be reported as median (IQR or range) instead of mean (SD).

The following changes have been made in Table 1 and results.

8. Results: Is there any data on baseline comorbidities (e.g., ASA, CCI, ECOG), length of hospital stay, and overall perioperative complications?

Thank you for the suggestion. We added details of ASA in Table 1. CCI and ECOG were not recorded during hospital stay. We also added the frequency of non-lymphatic peri-operative complications in Table 2. Clavien-dindo grade 3 or higher was counted in the analysis, as grade 1 and 2 complications were extremely frequent after RC.

9. Results: Among those who responded well to LE, what was the trend of lymphatic drainage before and after embolization?

We have added Figure 3 to better illustrate this trend. Both the clinical success and clinical failure group showed a visible decline in drainage volume after LE. However, pre-procedural drainage volume was higher in the clinical failure group, and the clinical success group had more patients who removed their drains, while only a few patients in the clinical success group removed their drain within 5 days after LE (85.7% vs 23.5%). We have added this detail in Figure 3 and line 210-214.

10. Discussion: The data reported on line 212 (77.6% successful drain removal) is a bit confusing.

Thank you for pointing this out. We rephrased the statement (line 246-248), to clarify that 77.8% of patients successfully removed their catheter within a week after LE.

11. Discussion: Please highlight the pros and cons of LE over conservative management:

We have added the role of LE compared to conservative management in line 255-260. We hope this helps clarify the pros and cons of LE.

11. Discussion: For those who are at higher risk for LE failure, what is your suggested strategy?

Refractory lymphatic leakage is difficult to manage. We suggest an early intervention for the high risk patients, as these patients would not respond well to conservative management. Although these patients also are at a higher risk of failure to LE, drainage volume still decreases significantly after LE (as shown in the added figure from question 9). We have further clarified this in out discussion (line 284-286).

**#Reviewer 2:**

1. I would appreciate a more detailed description of the embolization procedure, providing more technical information.

We described the LE process in further detail in line 134-144. Thank you for the suggestion

2. You only described perioperative variables of the cystectomy. Consider adding other variables, such as the quantity of embolizing fluid injected, etc.

Thank you for your suggestion. However, the quantity of embolizing fluid has not been recorded in our medical record and thus could not be evaluated. Still, we believe that procedure related risks would be minimal as all leaks were visually sealed during embolization.

3. For the multivariate analysis, why did you set 1000 mL as a cutoff? Is there a study that defines that value as a predictor of lymphatic leakage? You should consider using a discrete value, for example, every 100 mL/day, to understand how the probability of the event increases with each increment.

Thank you for the suggestion. We set 1,000mL/day as our cutoff value, as few previous studies illustrate drainage volume of 1,000 – 1,500 mL/day as a cutoff for surgical intervention. As mentioned in the manuscript there is no clearly defined volume for intervention in lymphatic leaks, and we believe that our study could approaching a cutoff value set for surgical management would be the proper starting point. We clarified this in our manuscript (line 177-179).

**#Reviewer 3:**

1. The authors should describe the PLND template in more detail.

Thank you for the suggestion. We described the PLND template in more detail, which is outlined in line 124-128 of the manuscript.

2. What is the proportion of patients with lymphoceles that underwent LE?

Thank you for pointing this out. As specified in our selection criteria, we intentionally excluded patients who underwent LE due to lymphoceles (line 103-105). First of all, we wished to focus on early leaks, which has few unique characteristics from lymphoceles (outlined in line 69-75). Moreover, most of the patients who presented with symptomatic lymphoceles were treated with sclerotherapy, thus did not undergo LE.

3. The authors state that (page 6, line 106): “Lymphatic leakage was diagnosed when radiographic evidence of leakage was found in lymphangiography“. Did all patients undergo lymphangiography?

Thank you for your question. Lymphangiography was used during all LE procedures to visualize leaks from the lymphatic vessel. Patients suspected of lymphatic leakage, and those who agreed on LE therefore underwent lymphangiography. When leaks were visualized, we could diagnose post-operative lymphatic leakage, and undergo LE. Patients without leaks shown in lymphangiography, although not included in the study, would not undergo LE. We clarified our manuscript accordingly (Line 111-114).

4. The authors excluded patients with evident lymphoceles on CT before LE. Why were those patients excluded? Why were not all patients included that underwent LE?

As specified in our selection criteria, we intentionally excluded patients who underwent LE due to lymphoceles. The main purpose of the study is to evaluate LE in early post-operative leaks. We further clarified our exclusion criteria to avoid any confusion (line 103-105). Thank you.

5. The size and extent of the lymphoceles are poorly characterized. Please elaborate.

Thank you pointing out. We described the characteristics of the lymphoceles that were found in 5 of our patients in line 225-229.

6. How was correct catheter placement during drainage of the lymphocele verified?

Thank you for pointing this out. A drainage catheter was placed in the pelvic space before closing the incision, and a post-operative abdominal x-ray was filmed, which confirmed its correct position. We added this description in line 130-132.

7. I suggest revising the statistics of the paper, adhering to the statistical outcome reporting guidelines provided by major urological journals

Thank you for pointing this out. We made adjusted corrections to our table.

8. How were the complications assessed? Was there a standardized approach?

All patients received imaging studies and out-patient follow-ups based on the National comprehensive cancer network bladder cancer guideline (version 4.2024). Image studies and out-patient records within 6 months after LE were reviewed for LE associated complications. We illustrated this in line 160-163.

9. The confidence intervals in the multivariable analysis are incredibly large.

Thank you pointing this out. We do acknowledge this as our limitation, and is mostly due to our small sample size. However, LE is yet an emerging therapy and our sample size is still one of the largest to be published. We believe that future studies with larger sample sizes would provide a clearer result.
