# Supplementary material for: Lymphatic embolization for early post-operative lymphatic leakage after radical cystectomy for bladder cancer
Source: PLoS One. 2024 Sep 24;19(9):e0305240. doi: 10.1371/journal.pone.0305240 (PMC11421775; doi:10.1371/journal.pone.0305240)
Supplement: Attachment — Submitted filename: Response to Reviewers.docx [file pone.0305240.s002.docx]

**Response to the editor:**

We would like to thank you for evaluating our manuscript and providing feedback to improve our paper. Here are our responses to your suggestion.

**1. I believe that, given the small sample size and multiple limitations of the study, the conclusions drawn may overemphasize the benefits of lymphatic embolization (LE). I suggest revising the conclusion section of both the abstract and the main manuscript body (line 323) to clarify that your findings are only suggestive of a possible effectiveness of LE in controlling post-operative lymphatic leaks after radical cystectomy.**

Thank you for your suggestion. We made the following changes in line 56-57 and line 322-323.

**2. The very high rate of clinical failure in the robotic surgical group warrants further elaboration. It appears that 3 out of 4 patients (75%) in this group experienced LE failure. This might suggest that the surgical team is less experienced with robot-assisted radical cystectomy (RARC) and typically performs surgeries in an open radical cystectomy (ORC) manner, potentially leading to more lymphatic manipulation and leaks (indicating a learning curve effect).**

We appreciate your response and concern regarding the high rate of clinical failure in RARC. RARC is becoming increasingly common in our practice, and is showing promising results in our institution as well. Unfortunately, this is a single-arm study with only 4 patients who received RARC. Thus, a direct comparison between ORC and RARC is difficult. When enough LE cases are collected, we would be able to attempt a study with a control group (conservative management or sclerotherapy vs LE), which would better elucidate the whether ORC or RARC might take a role in the clinical success of LE. Thank you.

**3. Another issue lies with the type of urinary diversion (UD). It was observed that 14 out of 45 patients (31.1%) underwent ureterocutaneostomy (UC), a simple UD associated with a much lower risk of lymphatic injury. However, UC is not commonly utilized in developed Western countries. This represents another limitation of your study, particularly in terms of the generalizability of your findings.**

Thank you for pointing out the high incidence of UC in our study. UC is carefully considered in patients with severe bowel conditions (IBD, radiation, bowel adhesions, etc.) in our institution, and avoided whenever possible. As a tertiary institution in Korea, patients with complicated bowel conditions are often referred specifically for the choice of UD in MIBC, therefore resulting in a higher rate of UC compared to ICUD or neobladders.

However, as lymphatic leakage is a complication associated with PLND rather than UD, we believe that the standard PLND template applied in all patients would support the generalizability of our study.

**4. The definition of clinical success needs to be properly referenced.**

In this study, we defined clinical success according to timing of drain catheter removal, re-intervention or post-procedural complications. This is unique to our study, and allows our study to truly represent patients who are at a higher risk of undesirable results after LE (further illustrated in line 290-299). We have further elucidated this is our Methods section (line 164-166). Thank you for the feedback.

**5. I encourage the respected authors to undertake a thorough linguistic and grammatical revision of the manuscript, ideally in collaboration with a native English-speaking physician. Several sentences do not clearly convey their intended meaning. For example, the sentence in lines 271-272, "Here, we found that a maximal preprocedural drainage volume exceeding 1,000 mL/day was an independent predictor of LE success," implies that a higher preprocedural drainage volume correlates with higher success, whereas the opposite is true. The term "success" should be changed to "failure" here.**

**Additional lines requiring significant grammatical and linguistic revisions include lines 71-75, 81-86, 103-108, 164-172, 183-185, and 294-300.**

Thank you for pointing out this issue. We went through a full revision of the manuscript to improve our linguistic errors accordingly.

**6. Lines 84-86 and 89-90 discuss similar concepts regarding the prevalence of lymphatic complications. I recommend removing one of these sections to improve the flow of the introduction. The introduction is generally good but needs a comprehensive grammatical and linguistic revision.**

We made the according change in our introduction (line 85-87), and also went through a thorough linguistic revision. Thank you for your suggestion.
